# Graves' Orbitopathy

**DOI:** 10.1155/2015/634234

**Published:** 2015-07-12

**Authors:** Yuji Hiromatsu, Jack R. Wall, George J. Kahaly, Hirohiko Kakizaki

**Affiliations:** ^1^Division of Endocrinology and Metabolism, Department of Medicine, Kurume University School of Medicine, Kurume, Fukuoka 830-0011, Japan; ^2^Department of Medicine, Nepean Clinical School, Nepean Hospital, The University of Sydney, Penrith, NSW 2751, Australia; ^3^Molecular Thyroid Research Laboratory, Department of Medicine I, Johannes Gutenberg University (JGU) Medical Center, 55101 Mainz, Germany; ^4^Department of Ophthalmology, Aichi Medical University, Nagakute, Aichi 480-1195, Japan

Graves' orbitopathy (GO) is an autoimmune disorder of the orbit that is closely associated with autoimmune thyroid diseases (AITD). Although the primary autoantigen(s) and precise mechanisms underlying the association between GO and AITD remain unclear, TSH receptors are thought to be the primary target of autoimmune reactions in GO patients. However, other antigens such as insulin-like growth factor 1 receptor, thyroglobulin, thyroid peroxidase, calcium binding protein calsequestrin, and collagen XIII are also involved in the orbital reactions in GO. This special issue is a great opportunity for the reader to learn the latest and emerging findings on the management of GO.

Recently the analytical performance and clinical utility of the functional thyroid stimulating autoantibodies (TSAb) and thyroid blocking autoantibodies (TBAb) have been extensively evaluated. In this special issue, E. Kampmann et al. performed a prospective study on the clinical relevance of the functional TSH receptor autoantibodies in a large collective of patients with GO. They noted that TSAb, not TBAb, were highly prevalent in severe and active GO. Serum TSAb levels correlated with all specific ophthalmic signs of the thyroid eye disease and mirrored the severity of GO. Thus, TSAb may be regarded as useful and reliable biomarkers for Graves' disease and associated GO.

The current first-line treatment for patients with severe and active GO is the administration of intravenous infusions of methylprednisolone pulses (IVMP). The European Group on Graves' Orbitopathy (EUGOGO) recommends single doses of 0.5 g of IVMP per day and a maximal cumulative dose of 8 g. In this issue, H. Eguchi et al. performed a retrospective study to look for risk factors of liver dysfunction during and after the IVMP therapy in a single center. Liver dysfunction was more frequently observed in males, in patients receiving high-dose methylprednisolone, and in patients aged over 50 years. Preexistent viral hepatitis was significantly associated with liver dysfunction (65% in patients positive for hepatitis B core antibody and patients positive for hepatitis C virus antibodies). Therefore, evaluation of preexisting risk factors and careful weekly monitoring of liver function during IVMP therapy and monthly thereafter for one year are warranted.

Also in this issue, M. Lin et al. introduced in a pilot and open study the subantimicrobial dose of doxycycline (50 mg daily for 12 wks.) for patients with moderate to severe and active GO. Eight of 13 patients showed improvement at 24 wks.; unfortunately this study lacks a control group. Since the subantimicrobial dose of doxycycline displays an anti-inflammatory and immunomodulatory function, it might serve as a new promising therapeutic strategy for GO. Future multicenter, double blind, randomized, controlled trials are therefore needed.

Orbital decompression surgery is indicated for rehabilitative reduction of the GO-induced exophthalmos and for restoration of the visual function in dysthyroid optic neuropathy. In this issue, N. Fichter and R. F. Guthoff performed a retrospective study and proposed that lateral wall decompression with orbital fat resection is the first choice in patients without disturbance of binocular functions and where moderate exophthalmos reduction is required.

Finally, the involvement of inferior rectus muscles (IRM) is a common but severe sequel in patients with GO. Recession of inextensible IRM is the first step in the correction of a restrictive hypotropia in GO. Y. Takahashi and H. Kakizaki performed a retrospective study to evaluate the predictive factors for the dose-effect relationship regarding unilateral IRM recession in GO. They found that the IRM thickness, the degree of intramuscular adipose changes, and the smoking status were relevant to it. Magnetic resonance imaging can detect both a thickened IRM and adipose change, enabling an accurate preoperative estimation.

In conclusion, the original articles in this special issue on Graves' orbitopathy provide new insights on diagnosis and management of GO. Future efforts to understand both the molecular pathology and mechanisms for the development of GO as well as to search for biomarkers of this complex disorder are indicated, and the performances of randomized clinical trials in patients with moderate to severe GO are keenly warranted.

*Yuji Hiromatsu* *Yuji Hiromatsu* *Jack R. Wall* *Jack R. Wall* *George J. Kahaly* *George J. Kahaly* *Hirohiko Kakizaki* *Hirohiko Kakizaki*

